# Supplementary material for: Mrp1 is involved in lipid presentation and iNKT cell activation by Streptococcus pneumoniae
Source: Nat Commun. 2018 Oct 15;9:4279. doi: 10.1038/s41467-018-06646-8 (PMC6189046; doi:10.1038/s41467-018-06646-8)
Supplement: Supplementary file 1 — Supplementary Information [file 41467_2018_6646_MOESM1_ESM.pdf]

SUPPLEMENTARY INFORMATION

Mrp1 is involved in lipid presentation and iNKT cell activation by *Streptococcus pneumoniae*

Chandra et al.

Supplementary Fig. 1: siRNA screen standardization, plate map and data analysis

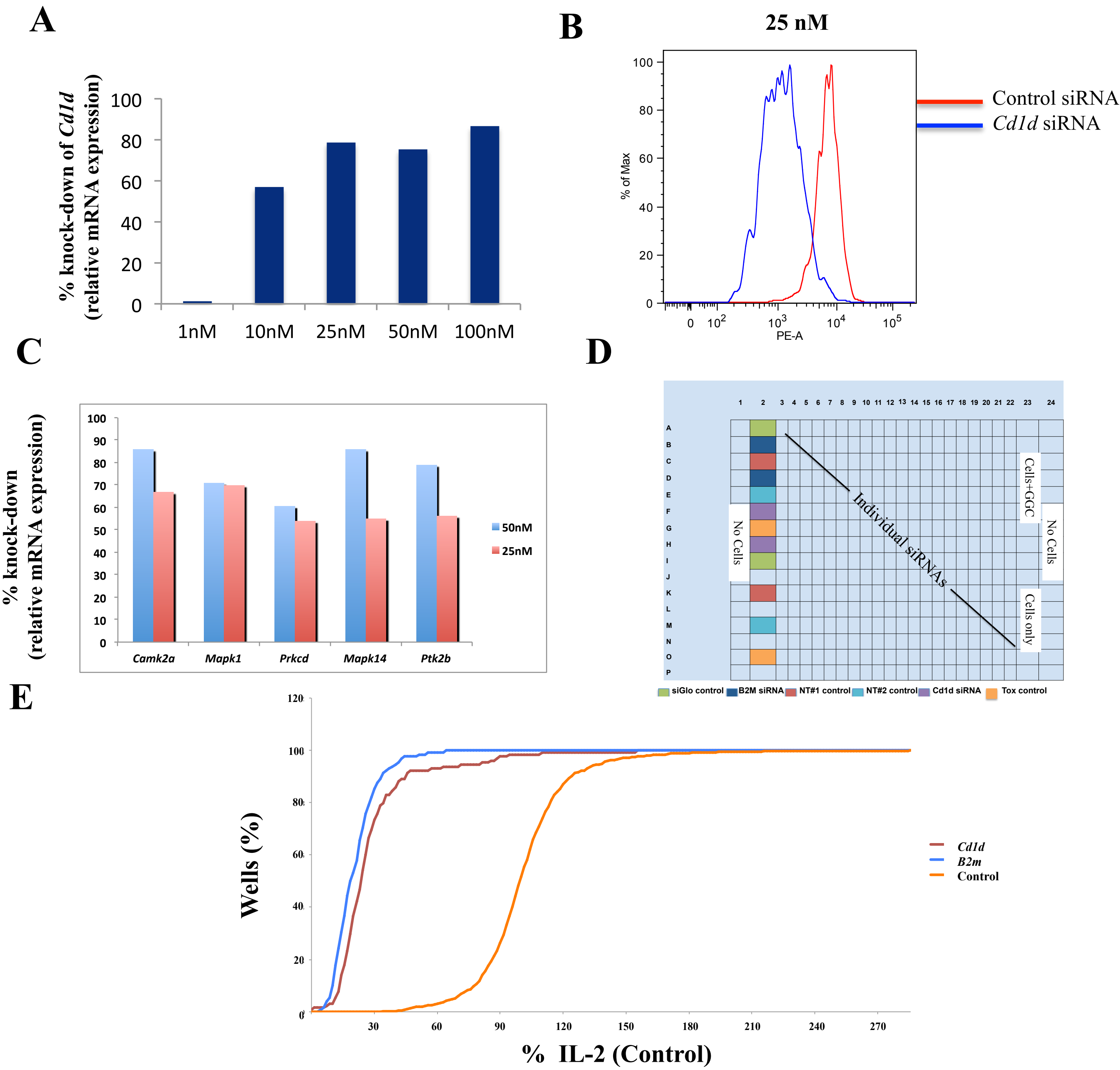

(A) J774-CD1d cells were transfected with indicated concentration of *Cd1d* siRNA pool. Figure represents % knockdown 36h after transfection in terms of relative mRNA expression compared to non-targeting control. (B) CD1d protein expression after knockdown in J774-CD1d cells at 25nM concentration of siRNA. (C) J774-CD1d cells were transfected with indicated siRNA for various kinase genes at the indicated concentrations. Figure represents % knockdown after transfection in terms of relative mRNA expression compared to non-targeting control. (D) Plate map used for the screen. It includes various positive and negative controls as indicated. siGLO = gives green color as control for transfection efficiency; NT= non targeting siRNAs; TOX = transfection control, to assess transfection efficiency based on cell death. 'Cells only' refers to J774-CD1d cells only with transfection reagent but no siRNA and 'Cells + GalGalCer' when antigen was also added but no siRNA. 'No cells' were background only wells containing media and 'cells only', iNKT hybridoma cells were added to each well.(E) Graph shows the separation between positive (*B2m* and *Cd1d*) and negative controls across 64 plates of the screen (tested in duplicate).

Supplementary Fig. 2: Network analysis of identified genes

A

Lipid antigen presentation, lipid metabolism and small molecule biochemistry network

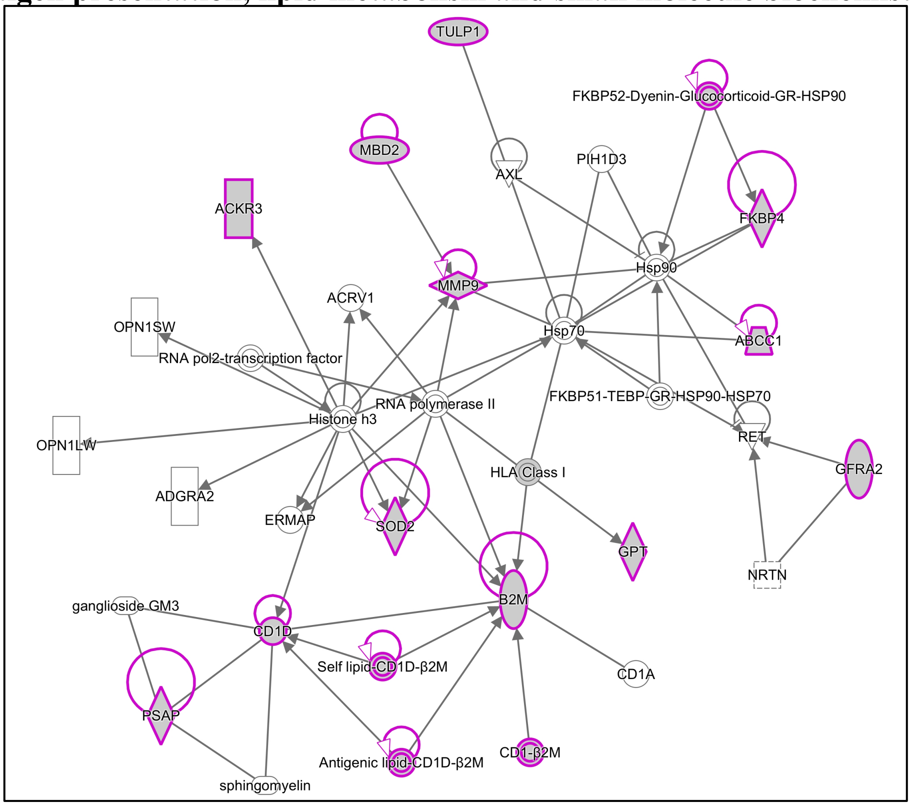

B

Cell morphology, cell-cell signaling and interaction network

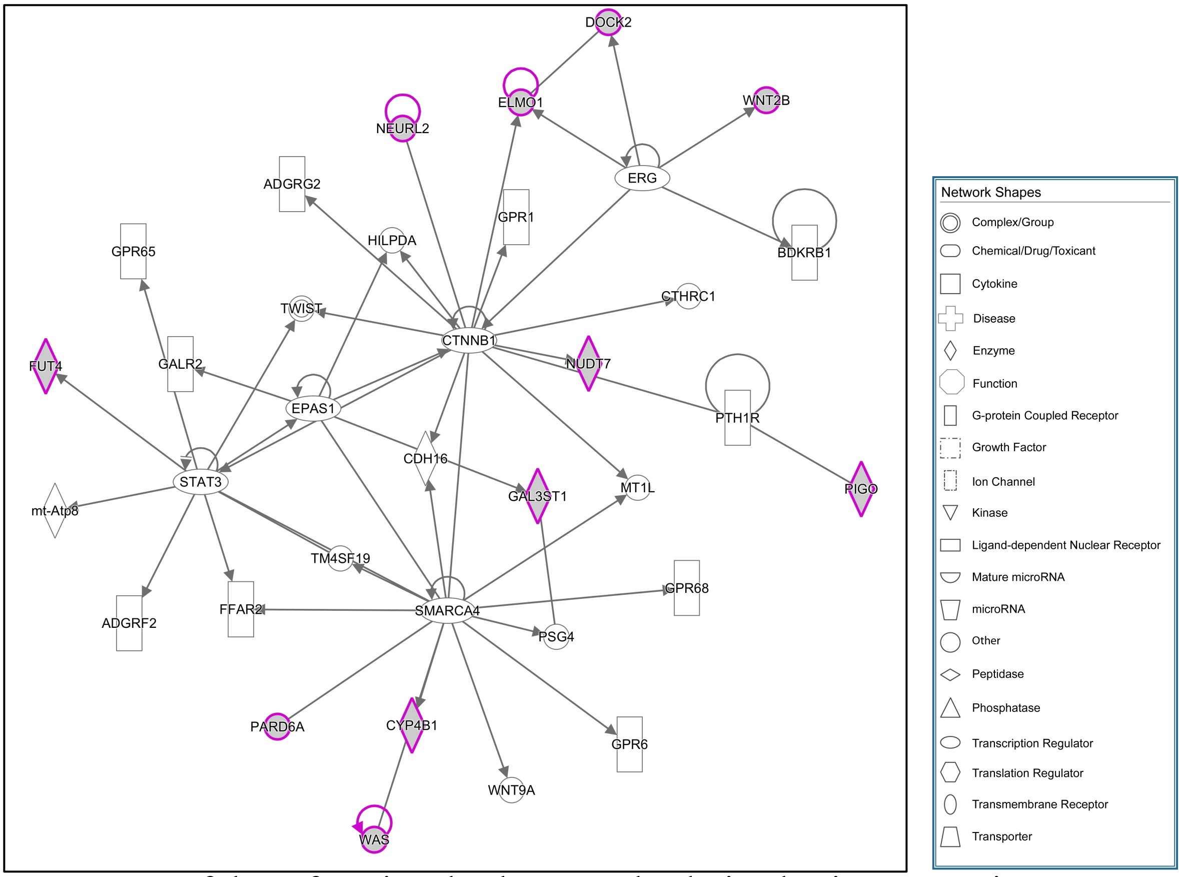

Figure represents two out of three functional sub-networks derived using Ingenuity Pathway Analysis. Only experimentally observed direct relationships were considered. Identified genes are highlighted.

Supplementary Fig. 3: Gene knockdown effects on MHC class I and class II protein expression

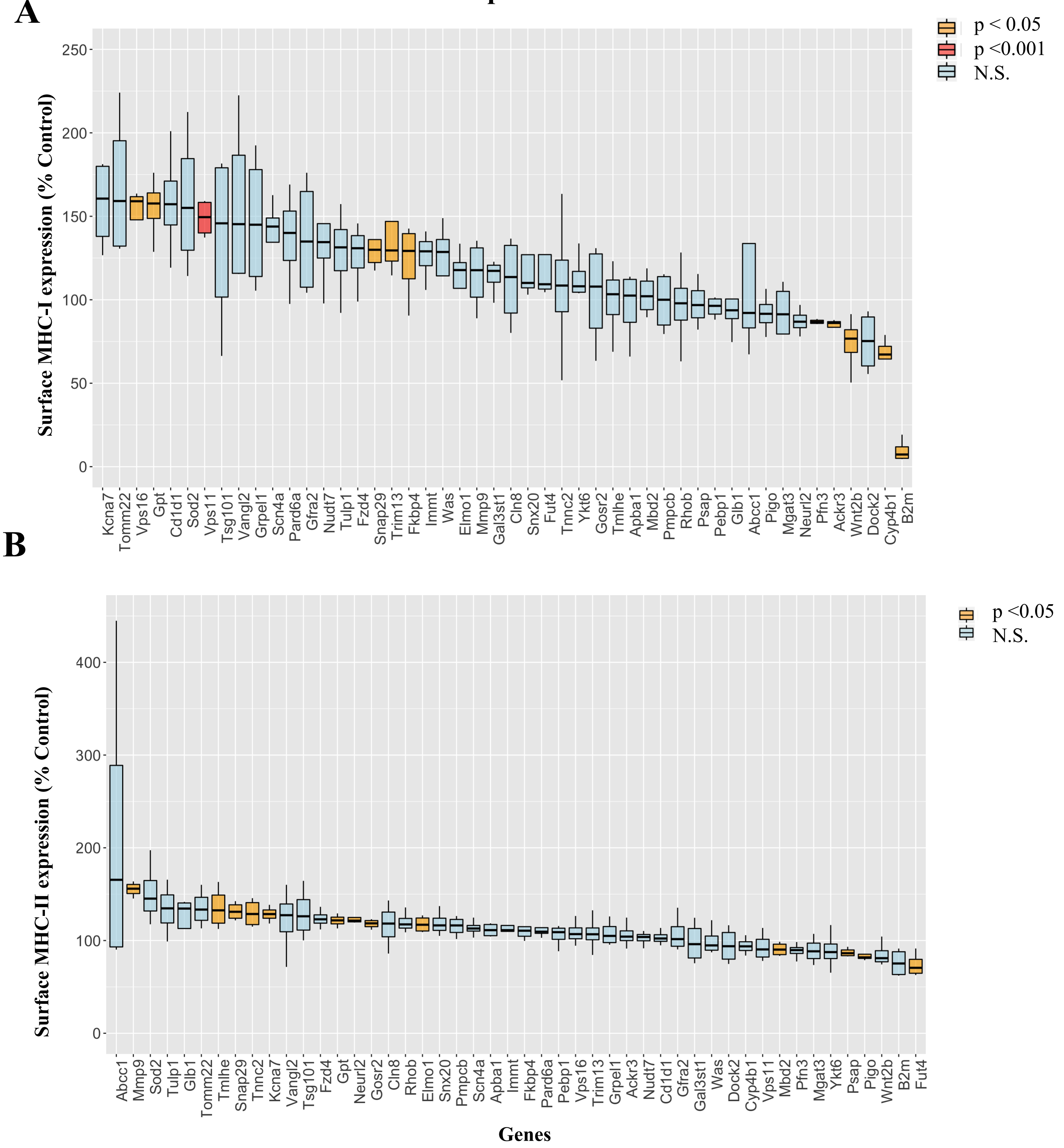

MHC class I (A) and II (B) expression was assessed on J774-CD1d cells (Live, Singlets) by flow cytometry following knockdown of 48 selected genes. The figure represents percentage change in mean fluorescence intensity (MFI) compared to non-targeting control. The average of four experiments is shown, p-values are indicated (paired t-test). Boxes represent the interquartile range and median, range of maximum and minimum values are displayed with vertical lines.

Supplementary Fig. 4: Gene knockdown effects on surface CD1d and antigen-CD1d complexes

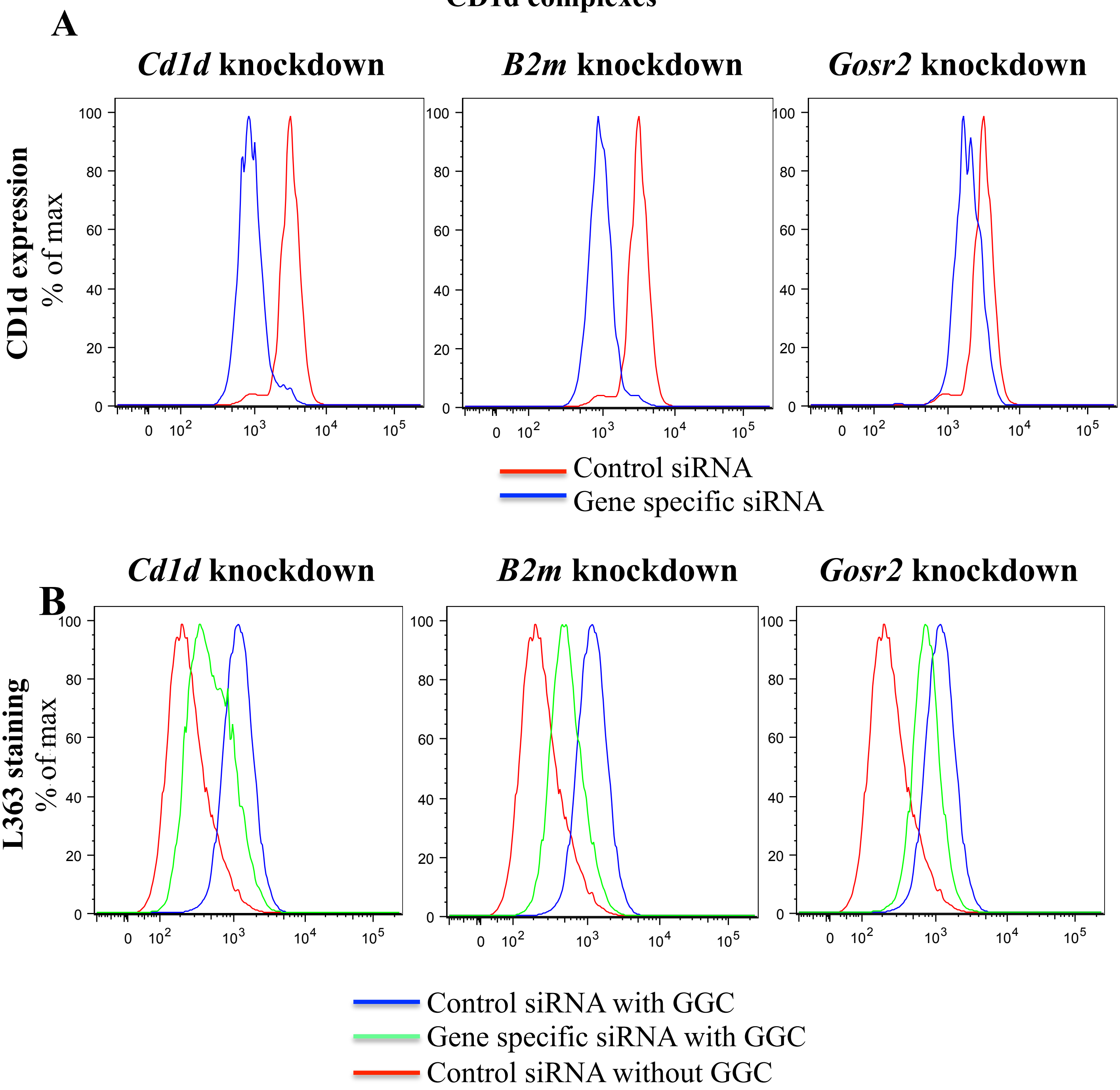

(A) Figure represents the decrease in surface CD1d expression by J774-CD1d cells after knocking down *Cd1d, B2m* or *Gosr2*. (B) Figure represents the decrease in surface CD1d-lipid complex expression by J774-CD1d cells after knocking down *Cd1d, B2m* or *Gosr2* and loading with GalGalCer. Representative data from one of four experiments.

Supplementary Fig. 5: Gene knockdown effects on MHC class II antigen presentation

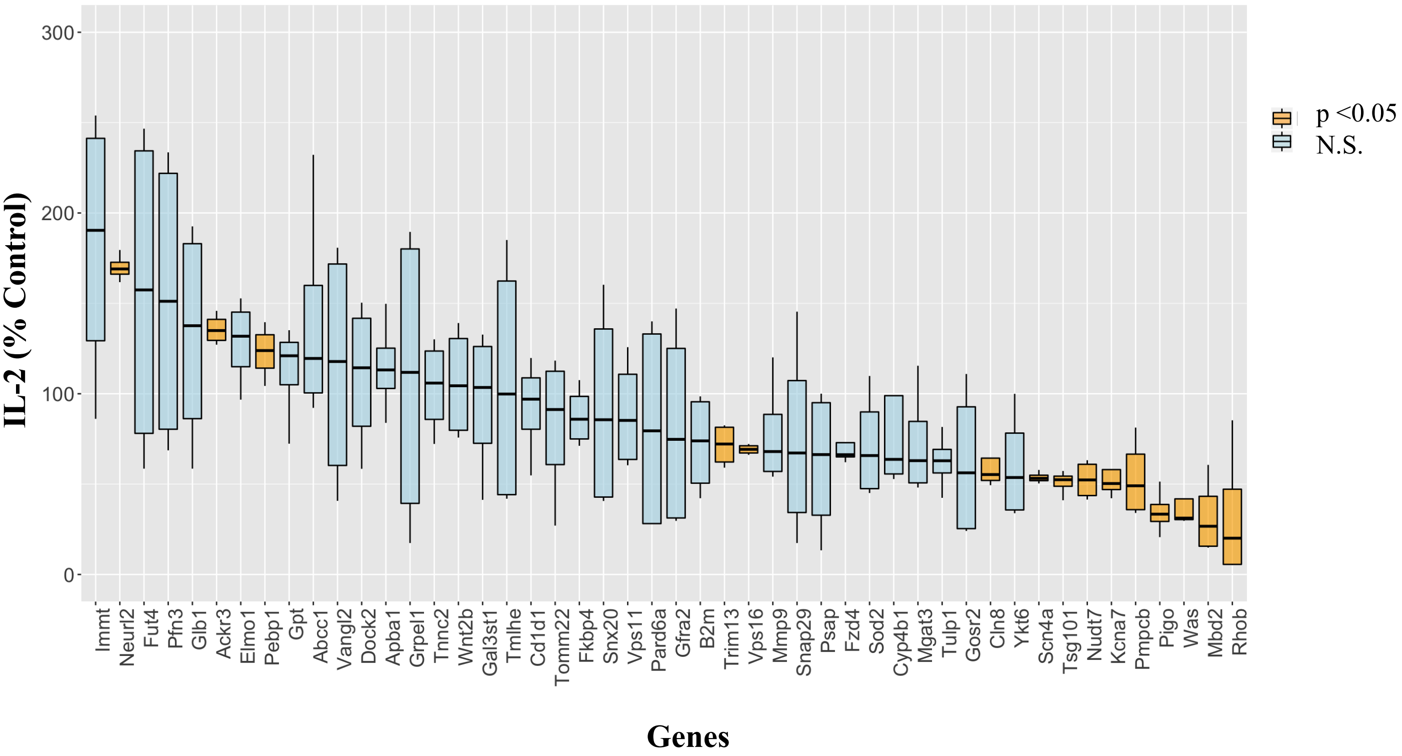

siRNA knock-down was performed in J774-CD1d cells for the indicated genes. 24h after transfection, cells were incubated with $CD4^+$ T cells isolated from DO11.10 TCR transgenic x *Rag*$^{-/-}$ mice for 48h in presence of ovalbumin. Supernatant was used to perform IL-2 ELISA. Figure represents the percent IL-2 as compared to non-targeting control. The average of two experiments is shown, p-values are indicated (paired t-test). Boxes represent the interquartile range and median, range of maximum and minimum values are displayed with vertical lines.

Supplementary Fig. 6: Gene knockdown effects on surface CD1d and antigen-CD1d complexes

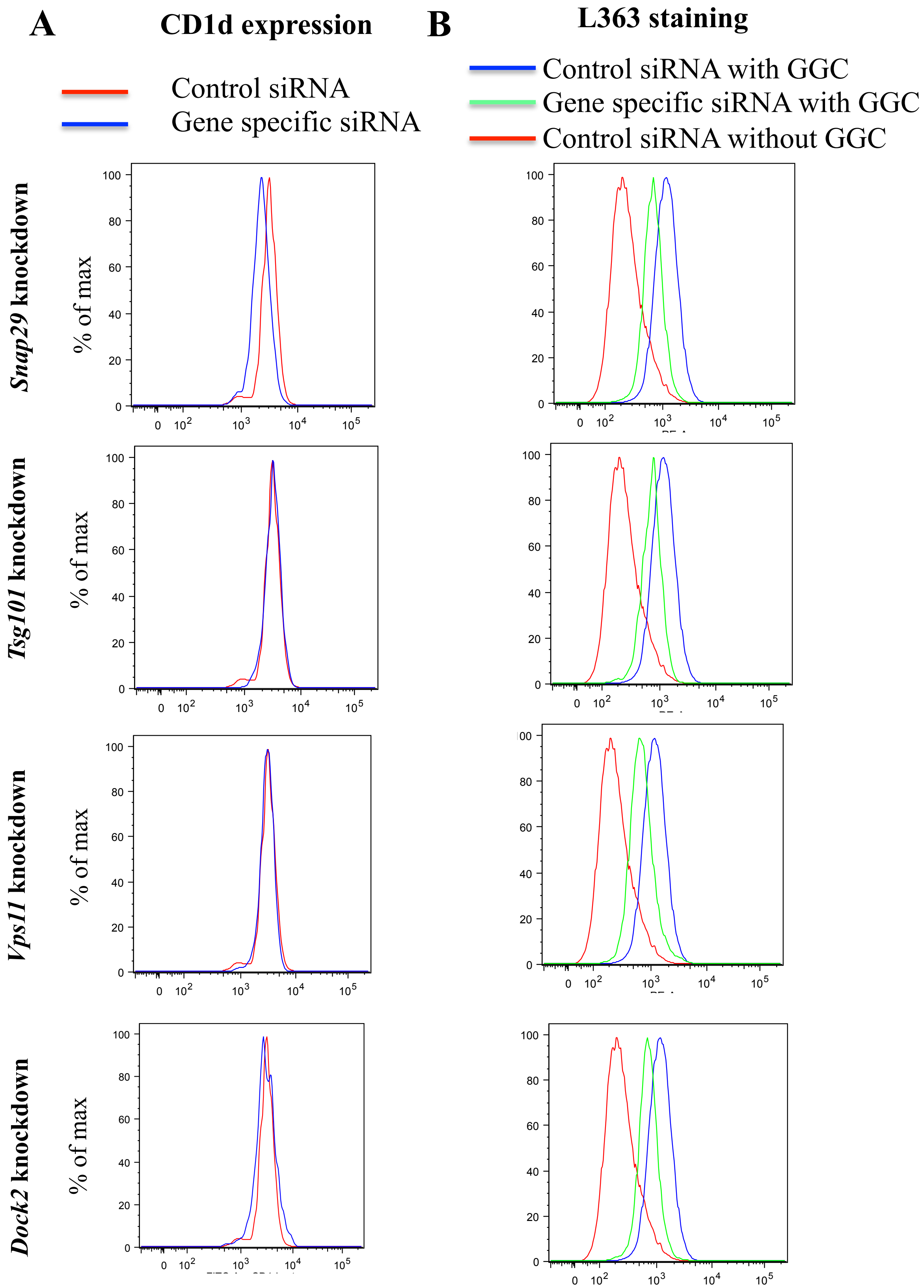

(A) Figure represents the decrease in surface CD1d expression by J774-CD1d cells after knocking down *Snap29, Tsg101, Vps11 and Dock2*. (B) Figure represents the decrease in surface CD1d-lipid complex expression by J774-CD1d cells after knocking down *Snap29, Tsg101, Vps11 and Dock2* after loading with GalGalCer. Representative data from one of four experiments.

Supplementary Fig. 7: Gene knockdowns at the protein level

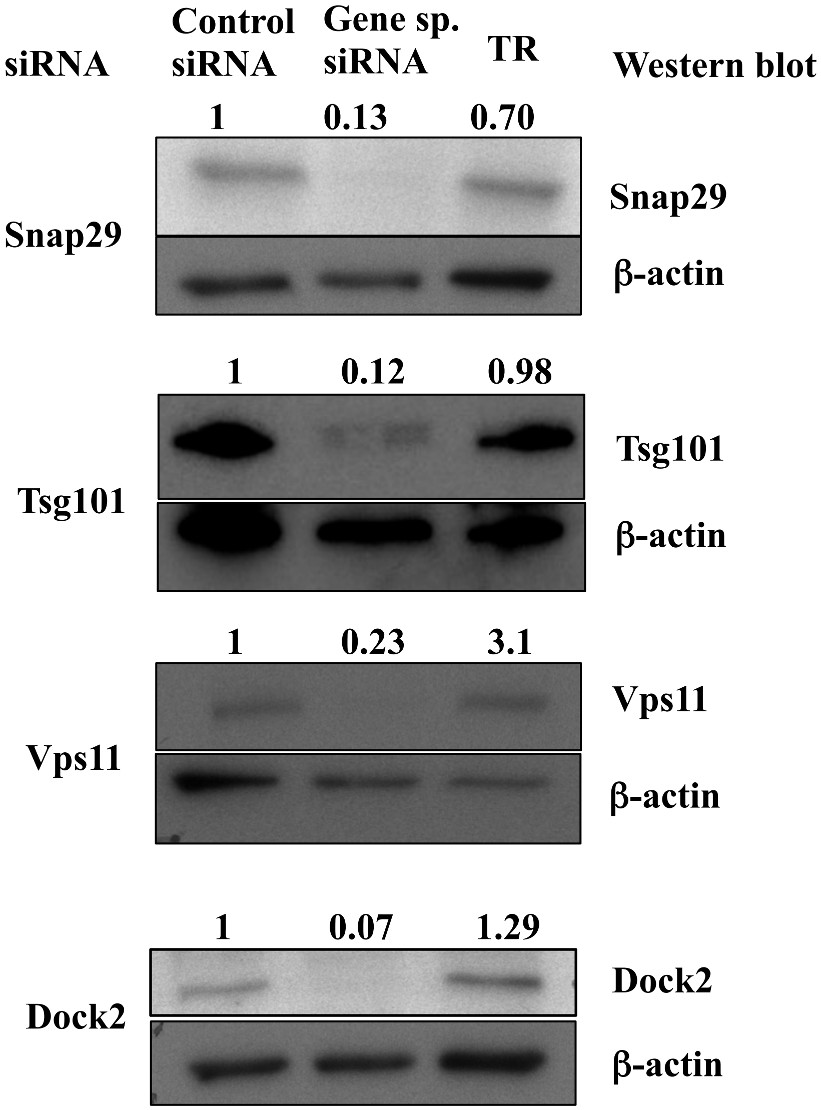

Figure shows the Western blot data obtained after transfection of J774-CD1d cells with control siRNA, gene specific siRNA or only transfection reagent (TR) for the indicated genes to monitor protein knock-down. 36h after transfection, cells were lysed and proteins resolved by SDS-PAGE followed by immuno-blotting against the target protein. Blots were stripped and re-probed for β-actin as the loading control. Values indicate the fold change in the band adjusted volume after normalizing it to β-actin. Quantitation was performed using Quantity One software.

Supplementary Fig. 8: Area and number of Lamp1^{+} vesicles in cells with gene knockdowns

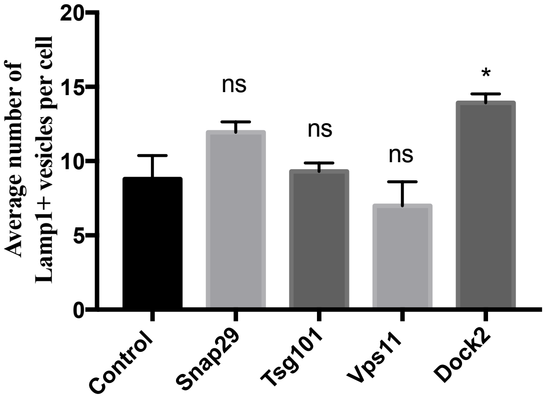

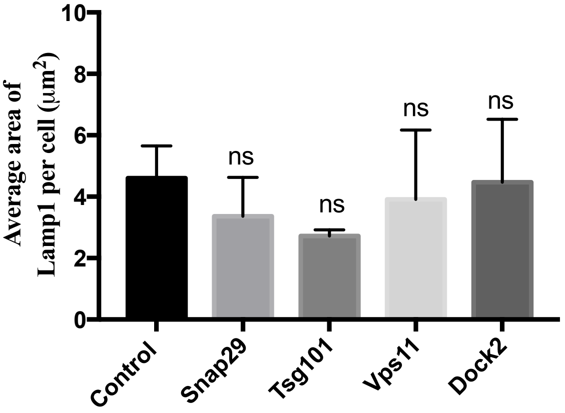

Figure represents the number of Lamp1 $^{+}$ vesicles and average area of Lamp1^{+} vesicles after knockdown of the indicated genes in J774-CD1d cells. Representative data from one of two experiments in which at least 200 cells per condition were analyzed. Graphs represent mean ± SEM. *$p < 0.05$ (one-way ANOVA).

Supplementary Fig. 9: Summary of effects of selected hits on CD1d localization and antigen trafficking

	Dock2	*Snap29*	*Tsg101*	*Vps11*
Block in early endosome	Yes	Yes	No	Yes
Lysosomal localization affected	Yes	Yes	No	Yes
Antigen traffic to lysosomes	No	Yes	Yes	No
	Cytoskeletal rearrangements	Membrane docking and fusion	Ubiquitinated cargos into MVBs/ESCRT-I complex	Vesicle trafficking to endosome/ lysosome

Figure represents the summary of CD1d localization in early or late compartments and antigen traffic to lysosomes after knocking down each of the four indicated genes.

Supplementary Fig. 10: : Gene knockdown effects on antigen presentation upon αGC stimulation

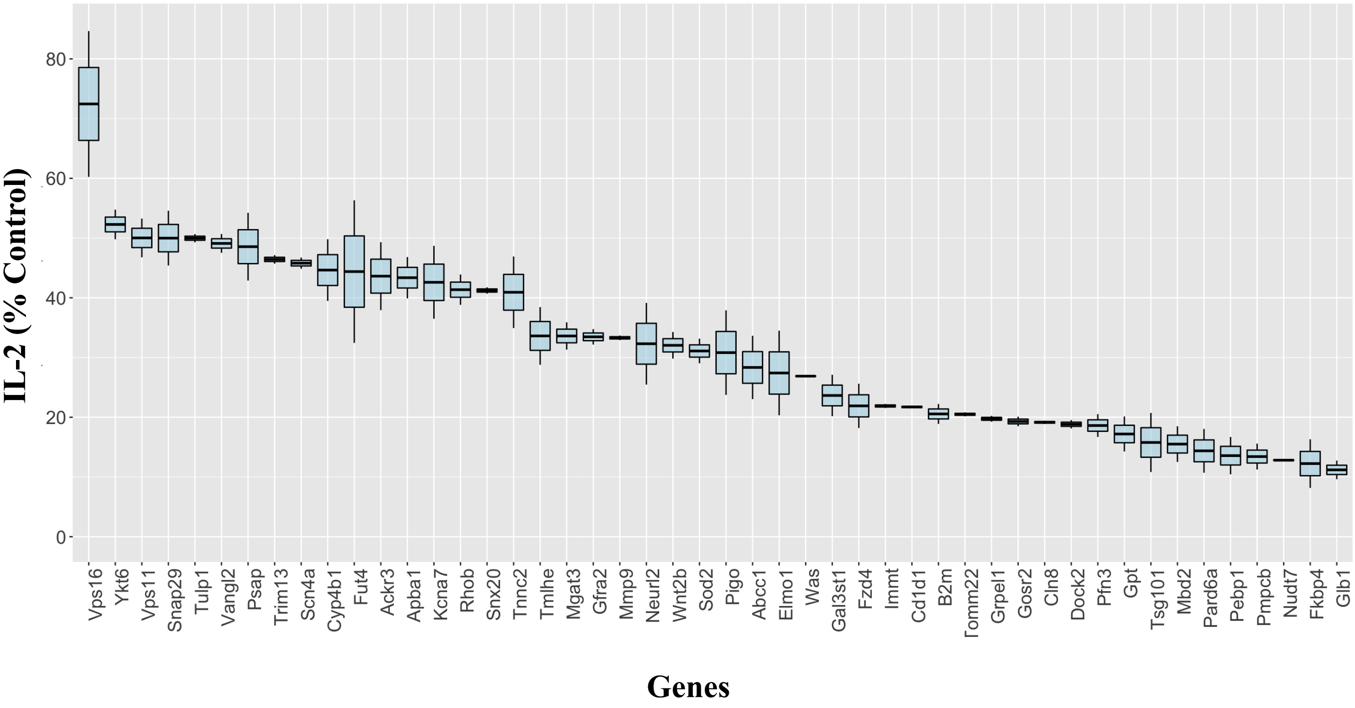

J774-CD1d cells were transfected with siRNA pools, 36h later these cells were loaded with αGalCer for 6h before culture with an iNKT cell hybridoma. 16h later supernatant was used to perform IL-2 Alpahlisa . Plot of normalized IL-2 release for selected hits compared to control. An average of two experiments is shown. Boxes represent the interquartile range; maximum and minimum values are displayed with vertical lines.

Supplementary Fig. 11: iNKT cell number in WT and *Abcc1*$^{-/-}$ mice

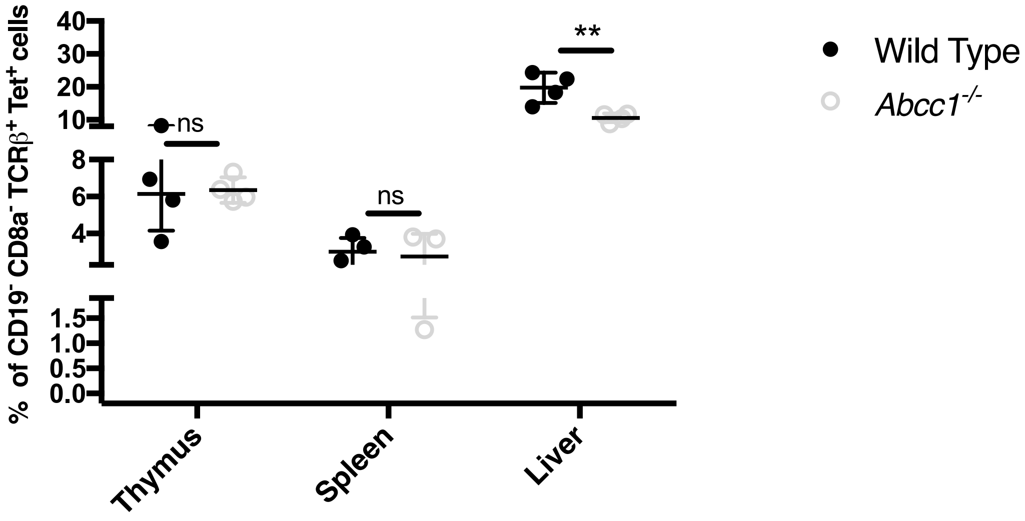

The figure represents the percentage of iNKT cells (live, singlets, CD19^{-}, CD8^{-}, TCRβ^{+}, Tet^{+}) cells in WT and *Abcc1*$^{-/-}$ mice in different tissues. The figure represents data from individual mice analyzed in two independent experiments. Graph shows mean ± SD. n = 4 for all tissues. **p < 0.01 (t test).

Supplementary Fig. 12: Gating strategy for iNKT cells

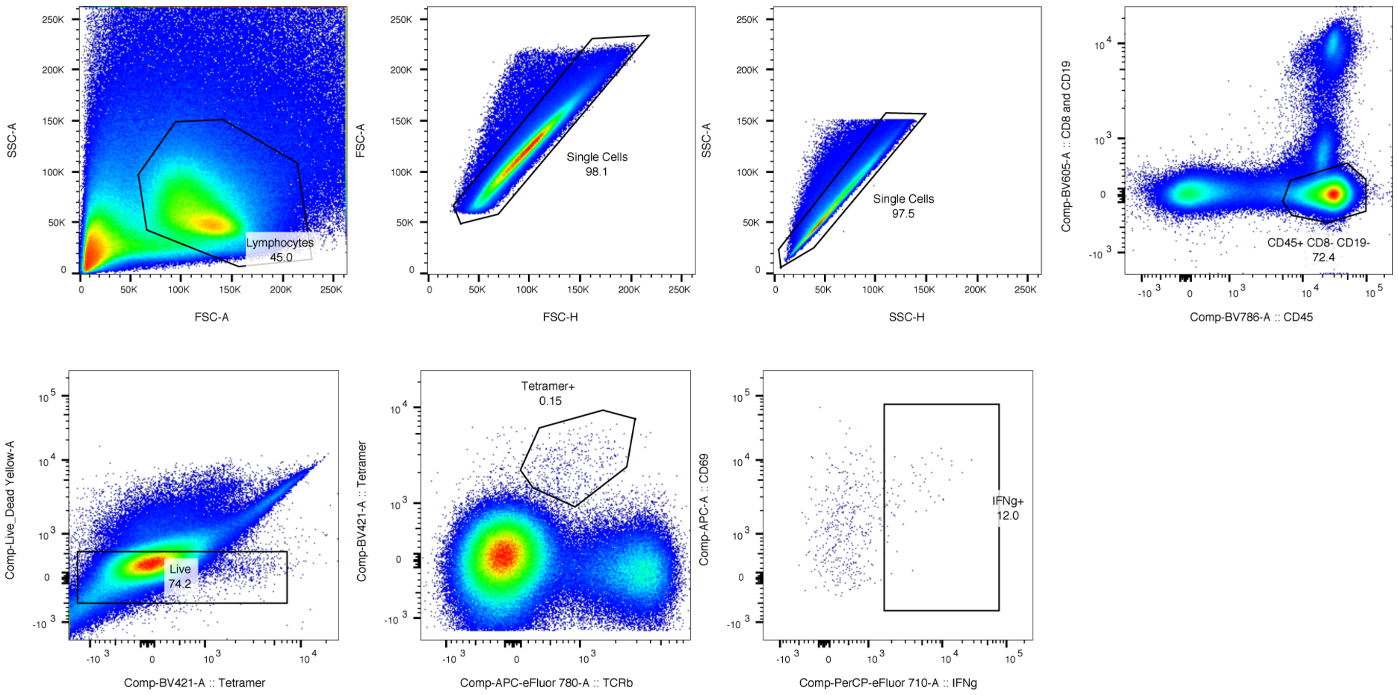

.

Figure represents the sequential gating strategy for analyzing lung iNKT cells (singlets, $CD45^+$, $CD19^-$, $CD8^-$, live, TCRβ$^+$, Tet^+). Gating method applies to data in Fig. 8F.

Supplementary Fig. 13: CD1d surface expression and antigen trafficking in *Abcc1*$^{-/-}$ knock-out mice

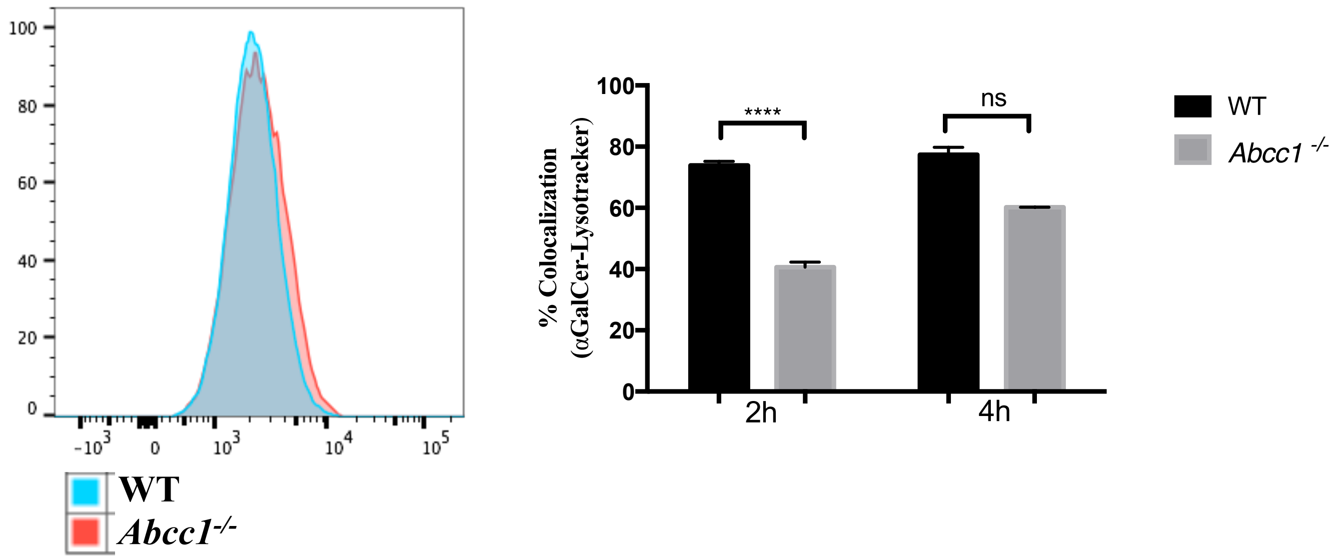

Peritoneal macrophages were isolated from wild-type (WT) and *Abcc1*$^{-/-}$ mice. (A) Live, Singlet, F4/80^{+} cells were stained with CD1d (B) Cells were incubated with αGalCer-Bodipy (2h and 4h) and LysoTracker (last 2h of chase). Images were acquired using confocal microscopy. Figure represents co-localization coefficients between αGalCer-Bodipy and LysoTracker. Representative figure from one of two experiments with at least 100 cells per condition. Graphs represent mean ± SD. ****$p < 0.0001$ (one-way ANOVA).

**Supplementary Table 1:** Table represents the selected GO classes and the corresponding genes for each class. Genes were classified using Bingo plug-in in Cytoscape.	
**GO class**	**Genes**
Transport	*Grpel1 Tsg101 Glra2 Grik5 Agxt Kcnk12 Timm9 Rhob Bc010801 Cat Vps16 Vps11 Kcng4 Ptger3 Cckbr Gif Lmbrd1 Was Elmo1 Tomm22 Cln8 Kcnh5 Snap29 Fkbp4 Elk3 Kcna7 Mip Best2 Pfn3 Best1 Agt Slc39a7 Hba-X Slc30a6 Acsl6 Apba1 Hspa9 Snx20 Eif4enif1 Psap Slc6a15 Fads2 Atp1a2 Slco1a6 Kctd4 Vav1 P2rx5 Slc16a3 Kcnj4 Tulp1 Prickle1 Gosr2 Abcc1 Slc15a4 Ykt6 Accn5 Scn4a*
Signal transducer activity	*Olfr1217 Gpr123 Gpr82 Glra2 V1rc11 Grik5 Olfr154 Vmn2r24 Nr2e1 Vipr2 Cxcr7 Olfr228 Cat Loxl2 Olfr513 Olfr461 V1rh3 Olfr1109 Olfr1284 Ptger2 V1ri10 Ptger3 Cckbr V1rc7 Tnfrsf17 Olfr623 V1rd13 Mrgprb8 Sh2d2a Cd163l1 Tomm22 Wnt9a Kcnh5 Olfr868 Bdkrb1 Elk3 Itgb6 Plch1 Trip13 Olfr954 Olfr549 Tas2r104 Olfr715 Cd5l Fzd4 Olfr790 Wnt2b Gpr153 P2rx5 Tulp1 Olfr606 Olfr905 Olfr685 Rgs6 Klre1 Gosr2 Olfr1022 Gfra2 Olfr688*
Vacuole	*Ncstn Psap Vps16 Cat Lmbrd1 Znrf1 Vps11 Ctbs Ttc3 Dnase2b Glb1*
Transporter activity	*Glra2 Grik5 Elk3 Kcna7 Kcnk12 Mip Best2 Pfn3 Best1 Slc39a7 Cat Hba-X Kcng4 Slc30a6 Slc6a15 Atp1a2 Slco1a6 Kctd4 P2rx5 Slc16a3 Kcnj4 Tomm22 Abcc1 Slc15a4 Accn5 Scn4a Kcnh5*
Carbohydrate metabolic process	*Mgat3 St3gal3 B3gnt1 Fut4 Parg Gpt Chst4 Car5a Ctbs Myc Pgm2l1 Glb1*
Peroxisome	*Dnm1l 0610009k11rik Nudt7 Cat Agxt Pex10 Acsl6*
Lysosome	*Ncstn Psap Vps16 Cat Lmbrd1 Znrf1 Vps11 Ctbs Dnase2b Glb1*
Endoplasmic reticulum	*Tmc6 Clstn2 Trim13 Fads2 Ube2j2 Pigo Cyp4b1 Ifrg15 Ncstn Rnf133 Lrat Pebp1 Gpt Gosr2 Ormdl3 Cat Rdh16 Cln8 Ykt6 Acsl6 Ormdl3 Cat Rdh16 Cln8 Ykt6 Acsl6*
Cytoskeleton organization	*Fmn2 Dock2 Pfn3 Ablim3 Nf1 Fbxo5 Neurl2 Cln8 Was Elmo1*
Lipid metabolic pathway	*Cyb5r1 Psap Thedc1 Fads2 Pisd Rdh1 Pigo Ugt1a10 Lrat Plch1 Liph Gpt Cat Rdh16 Cln8 Gal3st1 Acsl6 Ptdss2*
Calcium ion binding	*Tnnc2 Clstn2 Capsl Cetn1 Nid2 Actn3 C1s Mmp24 Anxa8 Efha1 Agt Plch1 Mmp1a*
Cellular component organization	*Grpel1 Mmp9 Ablim3 Fkbp4 Grik5 Cetn1 Rrm2b Nr2e1 Lats2 Dock2 Pfn3 Gtf2a1 Agt Timm9 B3gnt1 Fbxl11 Fbxo5 Pex10 Myc Chd4 Fn1 Pard6a Ccnk Dtx3l Nf1 Vangl2 Mbd2 Isl1 Was Vav1 Sod2 Elmo1 P2rx5 Hba-A2 Fmn2 Tulp1 Rnf2 Dnmt1 Cdc2a Neurl2 Cln8 Rere*
Mitochondrion	*Grpel1 Cyb5r1 Gfer Car5a Rrm2b Agxt Arl2bp Agxt2l2 Adck1 Lace1 Timm9 Bc010801 Cat Myc Slc30a6 Acsl6 Hspa9 Mrpl4 Dnm1l 1110020g09rik Hddc2 Psap 0610009k11rik Immt 1810015h18rik Pisd Sod2 Efha1 Tmlhe Acas2l Pebp1 Tomm22 Bik Pmpcb*
Organelle organization	*Grpel1 Ablim3 Cetn1 Rrm2b Lats2 Dock2 Pfn3 Timm9 Fbxl11 Fbxo5 Myc Pex10 Chd4 Ccnk Dtx3l Nf1 Was Elmo1 Sod2 Fmn2 Rnf2 Dnmt1 Cdc2a Neurl2 Cln8 Rere*

Supplementary Table 2

Disease	Gene
Lysosomal storage diseases	
Fabry	*Psap*
Gaucher	*Psap*
Gangliosidosis	*Glb1, Psap*
Metachromatic leukodystrophy	*Gal3st, Glb1, Psap*
Niemann-Pick	*Glb1, Gal3st*
Disorders of vesicles of lysosomal lineage: the Hermansky-Pudlak syndrome	*Tsg101, Vps16*
Tay-Sachs disease	*Psap*
Krabbe disease	*Glb1, Psap*
Infection	
Pneumoconiosis	*Tsg101*
Inflammatory conditions	
Diabetes mellitus	*Kcna7, Sod2, Cd1d, Elmo1*
Crohn's disease	*Snx20, Fkbp4, Fut4*
COPD	*Abcc1, Mmp9*
Cancer	*Abcc1, Apba1, Cyp4b1, Dock2, Fkbp4, Fut4, Gal3st, Gfra2, Immt, Mbd2, Mgat3, Mmp9, Nudt7, Pebp1, Pmpcb, Rhob, Sod, Tsg101, Was, Wnt2b, Ytk6, Cd1d*

Using Disease association (http://diseases.jensenlab.org/Search)

Table represents the known disease associations of the selected hits.
