# Supplementary material for: Mrp1 is involved in lipid presentation and iNKT cell activation by Streptococcus pneumoniae
Source: Nat Commun. 2018 Oct 15;9:4279. doi: 10.1038/s41467-018-06646-8 (PMC6189046; doi:10.1038/s41467-018-06646-8)
Supplement: Supplementary file 3 — Description of Additional Supplementary Files [file 41467_2018_6646_MOESM3_ESM.pdf]

Description of Additional Supplementary Files

Supplementary Data 1

Description: Genes identified in primary screen: Data represents the normalized IL-2 values (% control) and viability (% control).

Supplementary Data 2

Description: Secondary screen analysis: Data represents the normalized IL-2 values for primary and secondary screen.

Supplementary Data 3

Description: GO analysis of the hits identified: Data represents various GO classes identified in our screen along with the genes in each class obtained using Cytoscape 2.8.3.

Supplementary Data 4

Description: Deconvolution screen: Data represents the normalized IL-2 values for the four different siRNAs used for identified genes.
