# Supplementary material for: Mrp1 is involved in lipid presentation and iNKT cell activation by Streptococcus pneumoniae
Source: Nat Commun. 2018 Oct 15;9:4279. doi: 10.1038/s41467-018-06646-8 (PMC6189046; doi:10.1038/s41467-018-06646-8)
Supplement: Supplementary file 8 — Reporting Summary [file 41467_2018_6646_MOESM8_ESM.pdf]

nature research

Corresponding author(s): Mitchell Kronenberg

Reporting Summary

Nature Research wishes to improve the reproducibility of the work that we publish. This form provides structure for consistency and transparency in reporting. For further information on Nature Research policies, see Authors & Referees and the Editorial Policy Checklist.

Statistical parameters

When statistical analyses are reported, confirm that the following items are present in the relevant location (e.g. figure legend, table legend, main text, or Methods section).

n/a	Confirmed	
☐	☒	The exact sample size (*n*) for each experimental group/condition, given as a discrete number and unit of measurement
☐	☒	An indication of whether measurements were taken from distinct samples or whether the same sample was measured repeatedly
☐	☒	The statistical test(s) used AND whether they are one- or two-sided<br>*Only common tests should be described solely by name; describe more complex techniques in the Methods section.*
☐	☒	A description of all covariates tested
☐	☒	A description of any assumptions or corrections, such as tests of normality and adjustment for multiple comparisons
☐	☒	A full description of the statistics including central tendency (e.g. means) or other basic estimates (e.g. regression coefficient) AND variation (e.g. standard deviation) or associated estimates of uncertainty (e.g. confidence intervals)
☐	☒	For null hypothesis testing, the test statistic (e.g. *F*, *t*, *r*) with confidence intervals, effect sizes, degrees of freedom and *P* value noted<br>*Give P values as exact values whenever suitable.*
☒	☐	For Bayesian analysis, information on the choice of priors and Markov chain Monte Carlo settings
☒	☐	For hierarchical and complex designs, identification of the appropriate level for tests and full reporting of outcomes
☒	☐	Estimates of effect sizes (e.g. Cohen's *d*, Pearson's *r*), indicating how they were calculated
☐	☒	Clearly defined error bars<br>*State explicitly what error bars represent (e.g. SD, SE, CI)*

Our web collection on statistics for biologists may be useful.

Software and code

Policy information about availability of computer code

Data collection	N.A.
Data analysis	R, Cytoscape 2.8.3, Image Pro, Zen Imaging software, ImageJ, GraphPad Prism, Quantity One software and IPA

For manuscripts utilizing custom algorithms or software that are central to the research but not yet described in published literature, software must be made available to editors/reviewers upon request. We strongly encourage code deposition in a community repository (e.g. GitHub). See the Nature Research guidelines for submitting code & software for further information.

Data

Policy information about availability of data

All manuscripts must include a data availability statement. This statement should provide the following information, where applicable:

- Accession codes, unique identifiers, or web links for publicly available datasets
- A list of figures that have associated raw data
- A description of any restrictions on data availability

Microarray data are deposited in Gene Expression Omnibus (GEO) and the accession number is GSE109717. The authors declare that the main data supporting the findings of this study are within the article and its Supplementary Information files. Extra data are obtained from the corresponding authors upon request.

Field-specific reporting

Please select the best fit for your research. If you are not sure, read the appropriate sections before making your selection.

☒ Life sciences ☐ Behavioural & social sciences ☐ Ecological, evolutionary & environmental sciences

For a reference copy of the document with all sections, see nature.com/authors/policies/ReportingSummary-flat.pdf

Life sciences study design

All studies must disclose on these points even when the disclosure is negative.

Sample size	Sample sizes were based on previous experience in carrying out antigen presentation assays
Data exclusions	No data were excluded
Replication	All findings were replicated
Randomization	Control mice were age matched and of the same strain as the Abcc1 deficient mice For microscopy data
Blinding	For microscopy data, the analysis was carried out by a scientist blinded to the nature of the groups

Reporting for specific materials, systems and methods

Materials & experimental systems

n/a	Involved in the study	
☒	☐	Unique biological materials
☐	☒	Antibodies
☐	☒	Eukaryotic cell lines
☒	☐	Palaeontology
☐	☒	Animals and other organisms
☒	☐	Human research participants

Methods

n/a	Involved in the study	
☒	☐	ChIP-seq
☐	☒	Flow cytometry
☒	☐	MRI-based neuroimaging

Antibodies

Antibodies used

Antibodies used against various proteins were as follows: PE Rat Anti-Mouse CD1d (1:100, 553846), Mouse Anti-Mouse H-2K[d] (1:200, 553566), Purified Rat Anti-Mouse IL-2 (1:250, 554424), Biotin Rat Anti-Mouse IL-2 (1:1000, 554426), Biotin Rat Anti-Mouse CD8a (1:250, 553029), Biotin Rat Anti-Mouse CD19 (1:250, 553784), FITC Rat Anti-Mouse CD107a (LAMP-1) (1:100, 553793), BV786 Rat Anti-Mouse CD45 (1:200, 564225), Brilliant Violet 605™, BV605 Rat Anti-Mouse CD19 (1:200, 563148), BV711 Hamster Anti-Rat/Mouse CD49a (1:200, 564863) and PE Rat Anti-Mouse IL-17A (1:200, 559502) from BD biosciences. Anti-mouse CD8a Antibody (1:200, 100743), IFN gamma Monoclonal Antibody PerCP-Cy5.5 (1:200, 45-7311-82), αGalCer:CD1d Complex Monoclonal Antibody (L363) (1:150, 12-2019-82), TER-119 Monoclonal Antibody, Biotin (1:250, 13-5921-85), TCR beta Monoclonal Antibody, APC-eFluor 780 (1:200, 47-5961-82), Biotin CD24 Monoclonal Antibody (1:250, 13-0247-82) and CD103 (Integrin alpha E) Monoclonal Antibody (2E7), FITC (1:200, 11-1031-85) from ebioscience. Rat anti-mouse I-A/I-E Antibody (1: 200, 107622), Alexa Fluor® 700 anti-mouse CD4 Antibody (1:200, 100430), PE/Cy7 anti-human/mouse/rat CD278 (ICOS) Antibody (1:200, 313520) and APC anti-mouse CD183 (CXCR3) Antibody (1:200, 126512) from Biolegend, Rabbit Anti-Mouse Rab5 antibody (1:200, ab18211), Rabbit monoclonal to Tsg101 (1: 1000, ab125011), Rabbit polyclonal to Dock2 (1:1000, ab74659), Rabbit monoclonal to Vps11 (1:1000, ab170869) and Rabbit monoclonal to Snap29 (1:1000, ab138500) from Abcam.

	Goat anti-Rabbit AF488 secondary antibody (1:1000, A-11034) and Goat anti-Rat AF55 secondary antibody (1:1000, A-21434) from Invitrogen. Anti-rabbit IgG HRP-linked (1:2500, 7074) and Anti-mouse IgG, HRP-linked (1:2500, 7076) from Cell Signaling Technology.
Validation	Available with catalog number from manufacturer

Eukaryotic cell lines

Policy information about cell lines

Cell line source(s)	J774-CD1d, a mouse Cd1d1.1 transfectant of the J774A.1 (ATCC) macrophage cell line generated by our lab. The iNKT cell hybridoma DN3A4-1.2 was obtained from Mark Bix and Richard Locksley [J Immunol 161, 3271-81 (1998)].
Authentication	J774 CD1d ttransfectants were tested periodically for CD1d expression and NKT hybridoma cells were tested for TCR expression using CD1d tetramers loaded with antigen.
Mycoplasma contamination	J774 antigen presenting cells were tested for mycoplasma by routine DAPI staining and microscopy analysis
Commonly misidentified lines (See ICLAC register)	None

Animals and other organisms

Policy information about studies involving animals; ARRIVE guidelines recommended for reporting animal research

Laboratory animals	WT (FVB) parental strain and Abcc1-/- mice were purchased from Taconic. All procedures were approved by the La Jolla Institute for Allergy & Immunology Animal Care and Use Committee. Mice of both genders were used and they were 8-12 weeks old
Wild animals	None
Field-collected samples	None

Flow Cytometry

Plots

Confirm that:

☒ The axis labels state the marker and fluorochrome used (e.g. CD4-FITC).

☒ The axis scales are clearly visible. Include numbers along axes only for bottom left plot of group (a 'group' is an analysis of identical markers).

☒ All plots are contour plots with outliers or pseudocolor plots.

☒ A numerical value for number of cells or percentage (with statistics) is provided.

Methodology

Sample preparation	For staining of cell surface molecules, cells were suspended in staining buffer (PBS, 1% BSA, 0.01% NaN3) and stained with fluorochrome-conjugated antibody at 0.1–1 µg/107 cells for 15 min in a total volume of 50 µl. FcγR-blocking Ab anti-CD16/32
Instrument	BD LSRII and BD Fortessa was used for acquisition
Software	BD FACS DIVA software was used for acquisition and Flowjo was used for analysis
Cell population abundance	iNKT cells in Thymus is between 3-6% of total T cells.
Gating strategy	Gating strategy has been shown is Supplementary Fig. 12

☒ Tick this box to confirm that a figure exemplifying the gating strategy is provided in the Supplementary Information.
